# Commentary on Patient Advocacy and Research Priorities in Olfactory and Gustatory Disorders

**DOI:** 10.1007/s40136-023-00444-7

**Published:** 2023-02-17

**Authors:** E. M. Garden, A. Espehana, D. Boak, N. Gadi, C. M. Philpott

**Affiliations:** 1 Rhinology & ENT Research Group, Norwich Medical School, University of East Anglia, Norwich Research Park, Norwich, NR4 7TJ UK; 2 Fifth Sense, Barrow-in-Furness, LA14 2UA UK; 3 Anglia Ruskin Medical School, Anglia Ruskin University, Bishop Hall Ln, Chelmsford, CM1 1SQ UK; 4 Norfolk & Waveney ENT Service, The Norfolk Smell & Taste Clinic, Norwich, NR31 6LA UK

**Keywords:** Anosmia, Hyposmia, Olfactory disorders, Parosmia, Phantosmia, Advocacy

## Abstract

**Purpose of Review:**

This paper outlines the challenges faced by people with smell and taste disorders (SATDs) and why patient advocacy is crucial in addressing these. It includes recent findings in identifying research priorities in SATDs.

**Recent Findings:**

A recent Priority Setting Partnership (PSP) conducted with the James Lind Alliance (JLA) has been completed and the top 10 research priorities in SATDs determined. Fifth Sense, a UK charity, has been working alongside patient and healthcare professions to drive awareness, education and research in this area.

**Summary:**

Following the completion of the PSP, Fifth Sense have launched six Research Hubs to take forward these priorities and engage with researchers to carry out and deliver research that directly answers the questions raised by the results of the PSP. The six Research Hubs cover a different aspect of smell and taste disorders. Each hub is led by clinicians and researchers recognised for their expertise in their field, who will act as champions for their respective hub.

## Introduction

### Smell and Taste Disorders

Smell and taste are often the forgotten senses, with their value only being recognised when they are gone. Smell and taste disorders (SATDs) include a range of symptoms: anosmia (complete lack of smell), parosmia (smell distortions), phantosmia (smell hallucinations) and dysgeusia (taste disturbances).

Despite the lack of awareness surrounding these senses, SATDs are common. European estimates suggest 19% of adults suffer from olfactory dysfunction, with this figure rising to 80% in those over the age of 75 [1]; thus, making it more profound than hearing loss or blindness in the UK. True taste loss is rare, but 60% of SATD patients report experiencing a loss of flavour [2]. Regarding gender difference, women present more readily with SATDs, but objective smell tests suggest that men appear to be worse affected [3–5].

Key causes of SATDs include sinonasal disease (62%), post-infectious olfactory disorder (PIOD) (11%), head trauma and idiopathic cases [6]. However, the SARS-CoV-2 pandemic has resulted in a changing prevalence of SATD, as > 60–75% of those infected experience anosmia [4, 7]. Although the majority recover within 4 weeks of onset, current data indicates that 10% have continued SATDs without spontaneous recovery [8]. Long-COVID data suggests that smell loss is the third most common symptom people are left with, while taste loss is the eighth [9].

Loss of one or both conditions has been associated with a significant reduction in physical and mental wellbeing, in addition to impacting everyday activities [10]. Individuals living with SATDs report high rates of depression (49%), anxiety (47%), impairment of eating (95%), isolation (64%) and relationship difficulties (59%) [11]. Recent studies have also identified anosmia as an independent risk factor for decreased life expectancy, due to anosmia acting as a marker for cumulative toxic environmental exposure [12]. Despite this, individuals suffering from SATDs find difficulties in accessing the required medical support, and often perceive that their illness is not taken seriously [13•]. Our recent study (*n* = 673) showed there is a wide variation in clinical practice; 76% reported their condition was recognised by an otolaryngologist, 64% by GP and 47% by neurologists [14]. Furthermore, patients are given little consistent information by clinicians regarding treatment or prognosis. This is likely due to the clear lack of research and trials regarding treatment of SATDs, leaving clinicians uninterested and with no answers.

This indicates a real need for increased recognition of the wider impacts of SATDs across society, widespread education and training for healthcare professionals, more specialist healthcare services and increased research into new treatments. We need a change in understanding of the significant role that smell and taste plays in our health, safety and wellbeing.

## Fifth Sense

Fifth Sense is the UK charity for people affected by SATDs; their vision is to transform society’s understanding of the importance of the senses of smell and taste, and through doing so transform the lives of those affected by SATDs. The charity provides support, information and a signpost to potential diagnosis and treatment to people affected by these conditions. They play a leading role in educating society on the importance of smell and taste to our lives and what it means to experience impairment of one or both senses.

### Chief Executive, Duncan Boak’s, Story

Duncan established Fifth Sense in 2012, in partnership with Professor Carl Philpott, Honorary Consultant Rhinologist and Director of the UK’s first dedicated National Health Service Smell and Taste Clinic, following his experience of losing his sense of smell due to a head injury in 2005, when he was 22 years old.

He found that losing his sense of smell had a significant impact on his experience and quality of life; affecting his relationships, mental health and how he formed memories. It also impacted his safety, having come very close to causing an explosion in his kitchen, which was full of gas that he was unable to smell. His GP at the time said that it was very rare, and that nothing could be done, he would just have to live with it. Consequently, he spent 6 years thinking that he was one of the only people in the world with this invisible condition, that no one else seemed to have heard of or know anything about.

Duncan was introduced to Professor Philpott, who had established his pioneering clinic the previous year. Following discussions together, Duncan had the idea to start the first charity in this area to address a significant and unmet need. Together, they established Fifth Sense to transform the way that SATDs are understood, recognised, treated and researched. Since then, Fifth Sense has grown and developed with significant input from members and has become an organisation that is recognised globally for its work; acting as a recognised, trusted platform that in turn engages and empowers others, and supports them to become advocates.

#### Volunteers and Ambassadors: Champions of Their Condition

Many Fifth Sense volunteers are themselves affected by a SATD. The charity supports them to be able to use their personal experience to support and educate others, and in return they receive validation and understanding, along with the satisfaction of playing an active role in the charity’s work. It can also bring a sense of community and acceptance, through having the opportunity to work as part of a team of people with the lived condition.

*I’d never met anyone else who couldn’t smell until I came across Fifth Sense. Getting involved has enabled me to be part of a community of people with a shared experience and understanding of the issues we all face.* Debs Davies, Fifth Sense Ambassador

Fifth Sense launched the Ambassador programme in 2020, at the height of the pandemic. Fifth Sense Ambassadors support the charities work in several ways, for example helping raise awareness and providing peer support. Carl lost his sense of smell following a head injury and helps increase awareness of the impact of anosmia amongst his colleagues in the police force.

I was speaking to someone who had a brain injury fifteen years ago and lost his sense of smell. In all that time no one had told him anything about it. I shared everything I knew and watched his eyes light up as I was able to answer some of the questions he had. Carl Hughes, Fifth Sense Ambassador

Nevertheless, advocates do not have to be personally affected by a SATD; there are passionate Fifth Sense Ambassadors within the fragrance, food and beverage industries who help raise awareness of the impact of olfactory disorders and the work that Fifth Sense is doing in their own fields.

Over the past few years I have increasingly become aware of people’s loss of smell and the challenges that this brings. I am very passionate about smell and I am delighted to have become an ambassador for Fifth Sense and be part of the charity’s work to support people with smell disorders and help people understand how important our sense of smell is. Louise Smith, founder of Wales Perfumery

#### Advocacy Through Partnerships

Fifth Sense is developing important collaborations with organisations in the public and private sectors which will help towards their goals and provide new opportunities for advocacy. Currently, they are working in partnership with Cadent, the UK’s largest gas distribution network, which is responsible for the physical supply of gas to the homes of around half of the UK population and play a significant role in safeguarding vulnerable customers. Natural gas is odourless and has an odorant—mercaptan—injected into it to warn people if it escapes. But what if you cannot smell it?

Cadent have recognised the safety and wellbeing implications of smell impairment and, together with Fifth Sense, they are working to highlight the essential role that the sense of smell plays in safety and wellbeing and enable people with olfactory disorders to stay safe and well at home. This important work is being driven by the experiences, concerns and needs of the charity’s members. In 2022, Fifth Sense launched its ‘SmellSafety’ survey to capture the safety concerns of people with olfactory disorders. Early results provide a revealing and stark insight into the challenges faced by the people we represent.

It is a constant worry following our move to a house with a gas cooker. My mother suffered from Alzheimer's and lived with us. On a couple of occasions she left the gas cooker on unlit. I have no sense of smell and our dog alerted me to something being wrong but even though I went in the kitchen I was not aware what it was. I returned to the lounge but the dog again barked at me and then in my face, with his paws on my knees. I called my young daughter and we followed the dog into the kitchen. My daughter screamed out as she realised the level of gas in the kitchen and we opened all the windows and back door. It was a very close call for our family. Thank god for the dog. Fifth Sense SmellSafety survey respondent

This partnership with Cadent will enable Fifth Sense to highlight the challenges associated with olfactory disorders in new ways and with new audiences.

#### Bringing Together Patients and Professionals to Transform Understanding

In order to increase mutual support, shared learning and increasing research in the field of SATDs, bringing together patients, clinicians and researchers have proved to be powerful forums. Fifth Sense has delivered a regular National Conferences since 2013, attended by patients, their partners and family members, along with clinicians and researchers. Patients get a better understanding of their condition from the experts present and learn about current treatment possibilities and research. These experiences can be transformational for people who, too often, have struggled to get information and support.

*I have had my condition for thirty-one years and I learnt more in seven hours than I have in those thirty-one years.* David Cherrie, 2019 Fifth Sense Conference attendee

In turn, clinicians and researchers get to hear first-hand the difficulties that people with this invisible disability face and are often moved and inspired to do more to help.

*Along with my team of clinical colleagues I have been fortunate enough to work with Fifth Sense on a number of patient information exchange days. This has enabled patients and clinicians to share good practice, challenges and frustrations with one another. This cooperative working is leading to a number of avenues of research to improve understanding and treatments in the future.* Mr Sean Carrie, Consultant ENT Surgeon, Newcastle Freeman Hospital

In 2022, Fifth Sense and the University of East Anglia partnered to deliver a 1-day symposium on SATDs at healthcare professionals, providing information on the treatment and management of patients. The event was held both in-person and online and attracted delegates from all over the world. At the forefront were volunteer Fifth Sense members, speaking candidly about how their condition has impacted on their lives, and impressing on the healthcare professionals in attendance on the need for empathy and support for patients.

*Excellent training day… it was really useful to have had a patient perspective on the day-to-day impact of smell disturbance.* Delegate at ENT Training Day at which Fifth Sense delivered a session in 2021

#### Representation and Campaigning for Societal Change

Patients face real challenges due to the lack of knowledge across the healthcare profession, and also the lack of recognition of the impact of SATDs across both the public and private sectors. This is where patient-focused organisations like Fifth Sense have a role to play, in advocating on behalf of the people they represent.

In 2020, Fifth Sense wrote to the General Medical Council, outlining how the significant numbers of people experiencing smell disorders due to COVID-19 meant that education and training of healthcare professionals needed to be improved. The GMC responded to this in 2022 by adding anosmia to their Medical Licensing Assessment content map, which sets out the core knowledge, skills and behaviours that medical trainees are assessed on to be ready to practice in the UK. This is a significant step forward in the goal of seeing widespread professional education on SATDs.

In January 2022, media reports highlighted how leading UK retailer Morrisons was telling consumers to use a ‘sniff test’ instead of relying on use-by dates on milk, as part of a drive to reduce food waste. Morrisons provided no suggestion as to what people with an olfactory impairment were supposed to do. Fifth Sense was contacted by large numbers of people expressing outrage that they were being excluded by this advice. Duncan Boak wrote to and met with Morrisons and the Food Standards Agency (FSA), representing the views and needs of the people we represent. There was a positive outcome to this, with the FSA updating the guidance on their website to include people affected by smell disorders and continuing to pass on Fifth Sense’s message to other food companies and retailers.

## Driving Patient Involvement in Research

The James Lind Alliance (JLA) is a non-profit initiative established in 2004 and now sits under the umbrella of the National Institute of Health and Social Care Research in the UK. Their aim is to identify and prioritise unanswered research questions through bringing together patients, carers and clinicians in Priority Setting Partnerships (PSP) [15, 16]. The key values underpinning the PSPs are (1) inclusion of a range of patient and clinician perspectives; (2) transparency of the methods, outcomes and vested interests of those taking part in the prioritisation; and (3) evidence-based knowledge—‘known unknowns’—on which to base prioritisation. The need for this was recognised due to a marked mismatch between the interventions that patients and clinicians wished to see compared with those being assessed by researchers [17].

The structured approach has continued to develop over time, with the JLA Guidebook (now in its 10th edition) detailing the structure [18]. A PSP can be initiated by anyone with a connection to a PSP, including healthcare professionals, patients, carers or researchers. A Steering Group is created which co-ordinates and implements the activity of the PSP. An initial survey is sent out to gather a longlist of research uncertainties; a literature search is conducted to determine what is already known, and an interim prioritisation survey is created. From the top-ranked questions in the second survey, a final priority setting workshop is held to generate a Top 10 list. The JLA then publishes both the long list and the Top 10; they take this forward to work with funders and researchers to influence their decisions on future research.

### Top 10

The PSP process was started with the formation of the Steering Group. The purpose of the Steering Group was to oversee the process and decide on the scope of the PSP. The group consisted of clinician specialists in rhinology, neurology, psychology and general practice. Non-clinical members include patients with SATD, carers of patients and a representative from JLA.

The Steering Group then agreed upon the scope for the PSP, meaning what research questions should be included. It was decided that questions regarding diagnosis, understanding pathophysiology, treatments and access to care in the UK would be included. Any questions asking about non-UK-based care and normal variations of smell and taste were excluded.

**Fig. 1:**
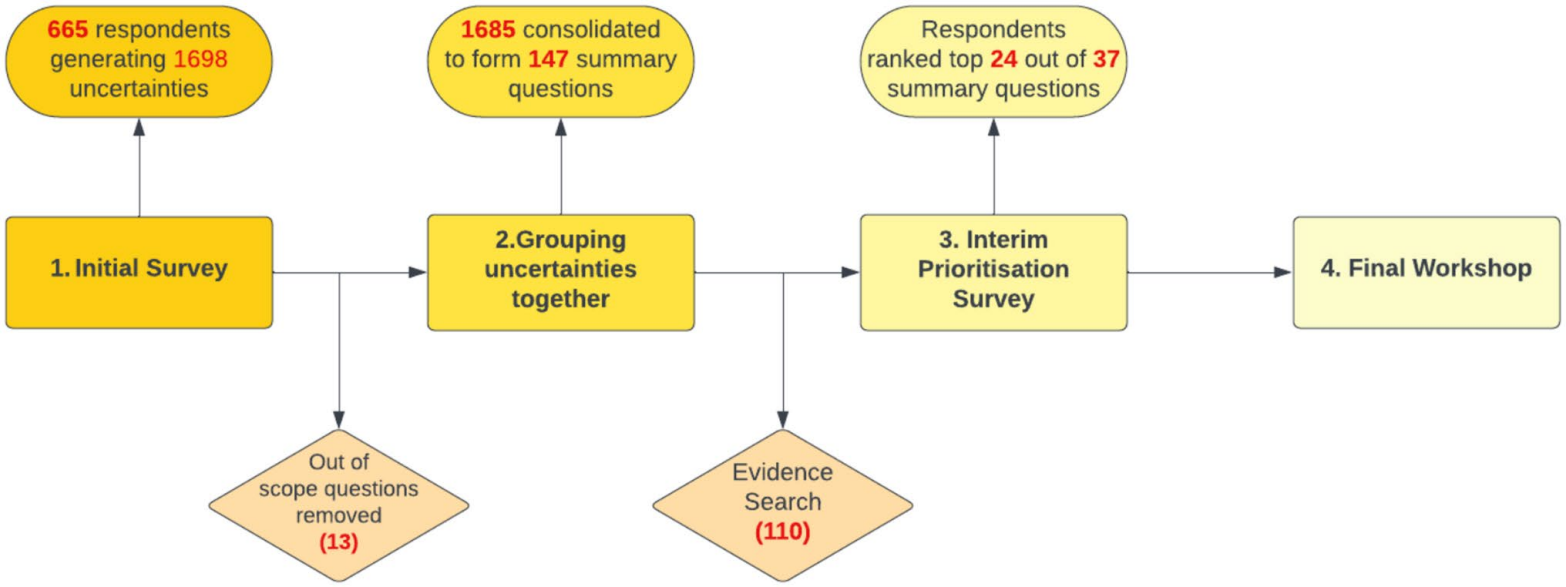
The PSP process

The process of the PSP is outlined in Fig. 1.

Through the PSP process established by the JLA, we have come up the top 10 research priorities in smell and taste disorders, which have been recently published by Fifth Sense, as seen in Table 1 [19, 20, 21••]. The full list of all 24 priorities agreed at the final PSP workshop can be found online at https://www.jla.nihr.ac.uk/priority-setting-partnerships/smell-and-taste-disorders/top-10-priorities.htm.

**Table 1:** Top 10 research priorities in smell and taste disorders [20, 21••]

1. How can we further our understanding of the mechanism of disease in the nerve pathways that affect smell and taste disorders, including where parosmia and phantosmia exist? 2. How can medical professionals be better educated in treating smell/taste disorders? 3. Do stem cells have the potential to treat smell and taste disorders? 4. How can regeneration of smell receptors be used to treat smell or taste disorders? 5. What are the mental health consequences of smell/taste disorders and how can these be managed effectively? 6. How can medical technology (e.g. implants) be used to rehabilitate sense of smell/taste? 7. How can the testing and investigations into smell/taste disorders be improved? 8. What role does genetics play in smell/taste disorders? 9. Are there any effective treatments for smell and taste disorders due to COVID-19 or any other viral infection? 10. What coping strategies help in dealing with smell/taste disorders?	

Overall, there was clear consensus reached on the final top 10 research questions during the PSP process. Given the current lack of understanding of the pathophysiology of SATDs, it is no surprise that mechanism of disease in the nerve pathways ranked most highly, with other aetiology-focused questions also appearing on the list. Fifty percent (5/10) of the questions related to supportive care and treatment: this seems to be a similar trend in other JLA PSP [21••, 22]. The list also includes questions quite specific to SATDs, such as stem cells and regeneration of smell receptors, which are new and emerging research areas in this field [21••]. Moreover, it is clear that stakeholders (patients) want more awareness and importance given to SATDs in the medical field, exemplified by the inclusion of priorities related to training professionals and diagnostic improvement. A temporal impact on the list can also be assumed: the initial survey occurred during the COVID-19 pandemic and 15% (*n* = 99) of respondents had a loss of taste or smell following COVID-19, potentially justifying the priority of smell loss due to COVID-19 making it into the top 10 [21••]. Digital health and technology are also very topical fields.

### Research Hubs

Through the publication of the top 10 research priorities, there were many discussions regarding what is lacking in research for smell and taste disorders. One of the most pertinent points that were raised was that while we know many of the causes of smell and taste impairment, we still do not fully understand the biology of normal smell and taste function, how these systems can become dysfunctional with injury or disease, or how to repair these systems when they are damaged.

Fifth Sense launched its Research Hub following the completion of the PSP. The goal of the Hub is to take forward the priorities identified through the PSP, engage and supporting researchers to carry out and deliver research that directly answers the questions that have been raised by the results of the PSP.

**Fig. 2:**
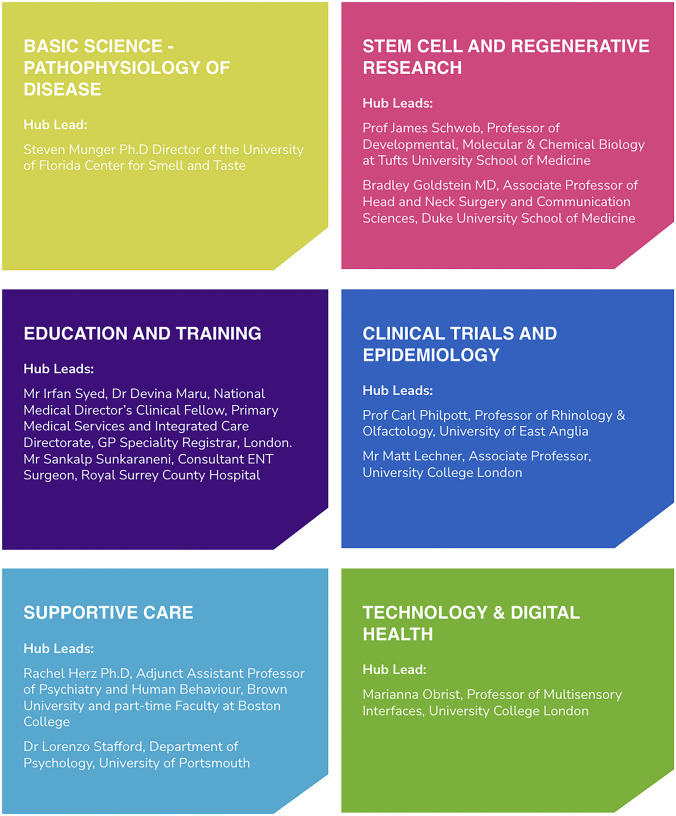
The Fifth Sense Research Hubs [23]

There are six Research Hubs in total, each covering a different aspect of smell and taste disorders. Each hub is led by clinicians and researchers recognised for their expertise in their field and will act as champions for their respective hub which can be seen in Fig. 2. Future work will involve developing more specific research questions within each respective hub, and continually monitoring the quality and quantity of research output to ensure this field no longer remains overlooked.

There six hubs are:

1. *Basic Science—Pathophysiology of Disease*

Led by Dr Steven Munger, Director of the University of Florida Center for Smell and Taste, it aims to answer questions relating to basic physiology of smell and taste and the pathophysiology behind the dysfunction of these senses.

2. *Education and Training*

Led by Mr Irfan Syed (Consultant ENT surgeon, University Hospital Lewisham), Dr Devina Maru (National Medical Director’s Clinical Fellow, Primary Medical Services and Integrated Care Directorate, GP Speciality Registrar, London) and Mr Sankalp Sukaraneni (Consultant ENT surgeon, Royal Surrey County Hospital), this hub aims to drive forward research and practice to improve education and training across the healthcare profession.

3. *Supportive Care*

Led by Dr Rachel Herz (Adjunct Assistant Professor of Psychiatry and Human Behaviour, Brown University and part-time faculty at Boston College) and Dr Lorenzo Stafford (Department of Psychology, University of Portsmouth), this hub will act as a focal point for researchers in the fields of psychology, dietetics, social sciences, complementary medicine and other relevant fields to develop nonmedical interventions for rehabilitation of smell and taste disorders including psychological and dietary.

4. *Stem Cell and Regenerative Research*

Led by Professor James Schwob (Professor of Developmental, Molecular & Chemical Biology, Tufts University School of Medicine) and Bradley Goldstein (Associate Professor of Head and Neck Surgery and Communication Sciences, Duke University School of Medicine), the main aim of this hub is to support and enable research that seeks to develop a better understanding of how the olfactory system can be regenerated through stem cell activation, drugs, biologics or other means.

5. *Clinical Trials and Epidemiology*

Led by Professor Carl Philpott (Professor of Rhinology and Olfactology, University of East Anglia), this hub hopes to engage with the National Institute of Health and Care Research (NIHR) and other funding bodies (Pharmaceutical, Medical Technology industries) to commission clinical trials and epidemiological studies in smell and taste disorders.

6. *Technology and Digital Health*

Led by Professor Marianna Obrist (Professor of multisensory interfaces, University College London), this hub aims to identify pathways to transform sensory care beyond the hospital through digital and technological innovation that helps to support the health and wellbeing of people with smell and taste disorders.

## Conclusion

As outlined in the introduction, people living with olfactory disorders face significant challenges in terms of recognition, access to specialist care and lack of treatment options. The COVID-19 pandemic has highlighted the global problem presented by smell disorders, and the wider health issues they create, for example the impact on diet, nutrition and mental health. A recently published study in the *British Medical Journal* suggests that due to COVID-19, there will be approximately 15 million and 12 million patients suffering from smell and taste dysfunction, respectively, worldwide, as of July 2022. The paper highlights that ‘health systems are unprepared for the challenge’ created by this situation [24].

Through the publication of the top 10 most important questions in SATDs, and establishing Research Hubs designed to answer these questions, we hope to bring patients together and enable their voices to be heard, continue working with clinicians, scientists, private and public sector organisations, and policymakers to effect change. More information can be found on the Fifth Sense website (https://www.fifthsense.org.uk/research/).

## Data Availability

Data sharing not applicable to this article as no datasets were generated or analysed during the current study.
